# Impact of donor human milk pasteurization methods on the gut microbiome of preterm infants

**DOI:** 10.1038/s41390-025-04386-2

**Published:** 2025-09-16

**Authors:** Claudia Ocampo-Chih, Hope Hendricks, Selin Weitkamp, Nidhi S. Gowda, Harinder Singh, Ritu Banerjee, Seesandra V. Rajagopala, Jörn-Hendrik Weitkamp

**Affiliations:** 1 Division of Neonatology, Department of Pediatrics, Vanderbilt University Medical Center, Nashville, TN USA; 2 Division of Pediatric Infectious Diseases, Department of Pediatrics, Vanderbilt University Medical Center, Nashville, TN USA; 3 Division of Infectious Diseases, Department of Medicine, Vanderbilt University Medical Center, Nashville, TN USA

## Abstract

**Background:**

Preterm infants are often fed donor human milk (DHM) when the mother’s own milk is insufficient or not available. Holder or Retort pasteurization is used to inactivate potential pathogens in DHM. The effects of DHM pasteurization methods on the infant gut microbiome are unknown.

**Methods:**

To compare the gut microbiome and clinical outcomes between preterm infants fed Holder- versus Retort-pasteurized DHM, we performed weekly collections of stool samples from infants born <34 weeks’ gestation and/or <1500 g birth weight. We analyzed stool samples from 150 patients exclusively fed DHM [Retort (*n* = 80), Holder (*n* = 54)] or exclusively fed mother’s own milk (*n* = 16). Whole-metagenome sequencing was performed to assess microbiome composition, diversity, and functional enrichment.

**Results:**

Compared to infants fed Retort-pasteurized DHM, infants fed Holder-pasteurized DHM showed higher alpha-diversity (Chao-1 *p* = 0.007) and a higher abundance of beneficial anaerobes, such as *Bacteroides thetaiotaomicron, Clostridium spp*., and *Bifidobacterium* *spp.* Functional enrichment analysis revealed significant differences in carbohydrate metabolism, transport systems, and regulatory pathways between feeding groups. There were no statistically significant differences in short-term clinical outcomes, such as necrotizing enterocolitis, length of hospitalization or death.

**Conclusion:**

Differences in pasteurization methods for DHM resulted in measurable gut microbiome changes in preterm infants.

**Impact:**

- It is known that the preterm infant gut microbiota is different in infants fed pasteurized donor milk compared to mother’s own milk. However, the impact of different pasteurization methods for donor milk on the infant gut microbiome is unknown.
- We show that the type of pasteurization of donor human milk influences the gut microbiome and its function in preterm infants.
- In contrast to feeding Retort-pasteurized donor human milk, feeding Holder-pasteurized donor human milk generates an infant gut microbiome similar to feeding mother’s own milk.

## Introduction

When mother’s own milk (MOM) is not available, preterm infants are often provided donor human milk (DHM), since, compared to formula, human milk is associated with improved rates of bronchopulmonary dysplasia (BPD) and necrotizing enterocolitis (NEC).^1–5^ The protective effects of human milk are largely due to immunomodulating compounds such as secretory IgA, lactoferrin, human milk oligosaccharides, leukocytes, cytokines, and growth factors that attenuate inflammatory responses.^6^ While DHM is the recommended alternative for nutrition of premature infants when MOM is not available,^7^ freezing and pasteurization results in an inevitable reduction of macronutrients and protective bioactive compounds.^8–12^ A recent review describes how various pasteurization methods reduce the risk for infection and highlights the importance of retaining immunological components such as antibodies and enzymes like lysozyme and bile salt-stimulated lipase.^13^

At present, pasteurized DHM is distributed from commercial and nonprofit milk banks that use different methods of pasteurization. The two most commonly used methods are retort processing, by which milk from large donor pools (200–400) is heated to 121˚ °C for 5 min at 15 PSI, and Vat or Holder pasteurization, which heats milk from smaller donor pools (1–10) to 63 ˚C for 30 min with subsequent rapid cooling and freezing.^8^ Retort-pasteurized milk is shelf stable and does not need refrigeration, which is an advantage for nurseries with limited resources for cooling and DHM storage. While it is known that lysozyme and sIgA (secretory immunoglobulin A) activity is lower in retort- versus holder-pasteurized DHM,^14^ there is limited data on the effects of different pasteurization methods on milk hormone levels and other factors that can influence the development of the infant gut microbiome.^15^ Like the effect of maternal health and dietary factors on the maternal–fetal microbiota axis, postnatal nutrition may influence the establishment of the neonatal gut microbiome with potentially long-term impact on health.^16^

Due to pandemic-related shortages in Holder-pasteurized DHM, the Vanderbilt University Medical Center (VUMC) Neonatal Intensive Care Unit (NICU) used Retort-pasteurized DHM from December 2019 to March 2022 but switched back to Holder-pasteurized DHM on April 4th, 2022. As part of a prospectively collected stool biorepository, we had access to stool samples from preterm infants fed these two different types of DHM in the same NICU, and we compared their gut microbiome and relevant clinical outcomes such as NEC, BPD, intraventricular hemorrhage (IVH), retinopathy of prematurity (ROP), mortality and length of hospital stay.

## Methods

### Study design and sample collection

We prospectively collected stool samples from infants admitted to the NICU at VUMC from July 2021 to December 2022. Stool samples were collected weekly from diapers or ostomies into ZYMO DNA/RNA Shield Fecal Collection Tubes and frozen at −80 °C until ready for analysis as described previously.^17^ Prospective stool collection began at birth once an infant was identified to be a candidate for study inclusion and continued until discharge or transfer to another hospital.

### Patient population and enrollment

This retrospective case-control study was approved by the Institutional Review Board at VUMC with a waiver of informed consent. Study data were collected and managed using REDCap.^18,19^ Inclusion criteria included patients <34 weeks’ gestation and/or birth weight of <1500 g, and ≤2 months postnatal age at the time of enrollment. Vanderbilt admits over 500 patients annually, who are fulfilling the inclusion criteria. Most infants in this population receive some form of DHM during their NICU stay. MOM is preferred and provided to the baby as soon as available. Infants <34 weeks do not receive formula in our NICU until 36 weeks PMA. When MOM is not available, DHM is offered following maternal consent as soon as the infant is considered stable enough to receive enteral nutrition. Patients were excluded if they had cyanotic congenital heart disease, abdominal wall defects, congenital diaphragmatic hernias or significant genetic conditions that were life-limiting or required patients to be NPO (nothing per mouth). Only infants exclusively fed DHM were included in this retrospective analysis. As a control, we included infants exclusively fed MOM. We only included infants with no or limited antibiotic exposure (e.g., maximal 48 h of antibiotics for possible early-onset sepsis). We analyzed one stool sample per patient once they received full volume exclusive DHM for at least 1 week. Demographic and clinical outcome data were collected from electronic medical records.

### Metagenomics sequencing

To analyze the microbial composition and functional potential, we performed whole-metagenome sequencing. Microbial DNA was extracted using the ZymoBIOMICS DNA Miniprep Kit (Zymo Research), followed by library preparation with the NEBNext DNA Library Prep Kit. Sequencing was conducted on an Illumina NovaSeq 6000 S4 platform, generating approximately 30 million paired end (2 × 150 bp) reads per sample.

### Assembly, classification, and dereplication of metagenome-assembled genomes

Metagenomic assembly, binning, and species-level clustering were performed using VEBA v1.2.0, incorporating within-SLC orthology analysis.^20,21^ Individual metagenomes were assembled per sample using the SPAdes v3.15.2 metaSPAdes mode.^22^ Prokaryotic binning was performed using MaxBin2 v2.2.7,^23^ Metabat2 v2.15,^24^ and CONCOCT.^25^ Non-redundant set of bins were obtained using the DASTool.^26^ Taxonomic classification was assigned using GTDB-Tk v2.2.3 with the R207 reference database,^27^ and quality assessment was conducted using CheckM v1.1.3 with the lineage_wf pipeline.^28^

To reduce redundancy, species were dereplicated using FastANI v1.32^29^ with a ≥ 95% ANI threshold, and connected components were determined using NetworkX v2.5 to generate species-level clusters (SLCs). For each SLC, pangenome proteins were further clustered into subspecies protein clusters using MMseqs2 with 80% query coverage and 50% identity.^30^ Prokaryotic gene models were generated with Prodigal v2.6.3 (--meta mode).^31^ Protein functional annotation was conducted using Diamond v2.1.7^32^ against UniRef90^33^ and KofamScan v1.3.0.^34^

### Gene Ontology (GO) enrichment analysis

Gene Ontology (GO) is a functional profiling method used to identify overrepresented biological processes, molecular functions, and cellular components in a set of genes or proteins. In this study, we applied GO enrichment analysis using the clusterProfiler package^35^ in R to compare the functional potential of the neonatal gut microbiome across three feeding groups: MOM, Holder-pasteurized, and Retort-pasteurized milk-fed neonates. The analysis focused on metagenomic functional annotations, particularly genes associated with carbohydrate metabolism, transport systems, and regulatory proteins.

### Statistical analysis

We calculated α-diversity, β-diversity, and differential abundance at the species level using Phyloseq,^36^ Vegan, and R version 4.3.2.^37^. Alpha diversity was assessed using observed species, Chao1 and Shannon indices, while β-diversity was evaluated using Bray–Curtis and Jaccard indices. Clinical data were analyzed using the Wilcoxon test for continuous variables and the Pearson test for categorical variables. Compositional data analysis was performed using ANCOM-BC2^38^ to identify differentially abundant taxa at the species level. Functional pathway enrichment analysis was conducted using the enrich function within the clusterProfiler v4.14.0 R package to perform KEGG pathway gene set enrichment analysis.^35^ Statistical significance was defined as *p* or *q* < 0.05 for all analyses.

## Results

Between July 2021 and December 2022, we collected weekly stool samples from 242 infants during the Retort-pasteurized DHM period and from 215 infants during the Holder-pasteurized DHM period. From these, 150 infants’ stool samples were selected for whole metagenome sequencing based on inclusion and exclusion criteria. This included 80 samples from the Retort DHM cohort, 54 from the Holder DHM cohort, and 16 from the MOM cohort. Infants were a mean of 28 (range 25–31) weeks’ gestation at birth and a mean of 4 weeks [0–14 weeks] old at the time of stool collection for microbiome analysis (Table 1). There were no significant differences in gestational age at birth, birth weight, sex, and race between groups. The C-section rate was slightly higher in the Retort group compared to the Holder group (82.5% vs. 64.8%, *p* 0.067). Importantly, antibiotic exposure prior to sample collection was similar in the Retort (81%) and Holder (78%) cohorts (*p* = 0.37).

**Table 1:** Demographics

	Retort (*n* = 80)	Holder (*n* = 54)	MOM (*n *= 16)	*P*-value Retort vs Holder	*P*-value
Gestational age, weeks	28.70 ± 2.91	28.37 ± 2.93	26.46 ± 2.09	0.530	0.020
Birth weight, kg	1.20 ± 0.42	1.10 ± 0.38	0.86 ± 0.20	0.160	0.010
Age (weeks), mean (range)	4.01 (0–14)	3.94 (0–12)	5.44 (2–13)	0.900	0.210
Antibiotic exposure within 4 weeks of stool collection, *n* (%)	65 (81.3)	41 (75.93)	3 (18.75)	0.598	<0.001
Mode of delivery, *n* (%)				0.033	0.068
Cesarean section	66 (82.5)	35 (64.8)	13 (81.2)		
Vaginal delivery	14 (17.5)	19 (35.2)	3 (18.8)		
Sex, *n* (%)				0.374	0.295
Female	34 (42.5)	28 (51.9)	5 (31.2)		
Male	46 (57.5)	26 (48.1)	11 (68.8)		
Race, *n* (%)				0.926	0.895
White	45 (56.2)	30 (55.6)	11 (68.8)		
Black	24 (30.0)	17 (31.5)	3 (18.8)		
Hispanic	8 (10.0)	4 (7.4)	1(6.2)		
Asian	2 (2.5)	1 (1.9)	1 (6.2)		
Unknown	1 (1.2)	1 (1.9)	0 (0.0)		

**Fig. 1:**
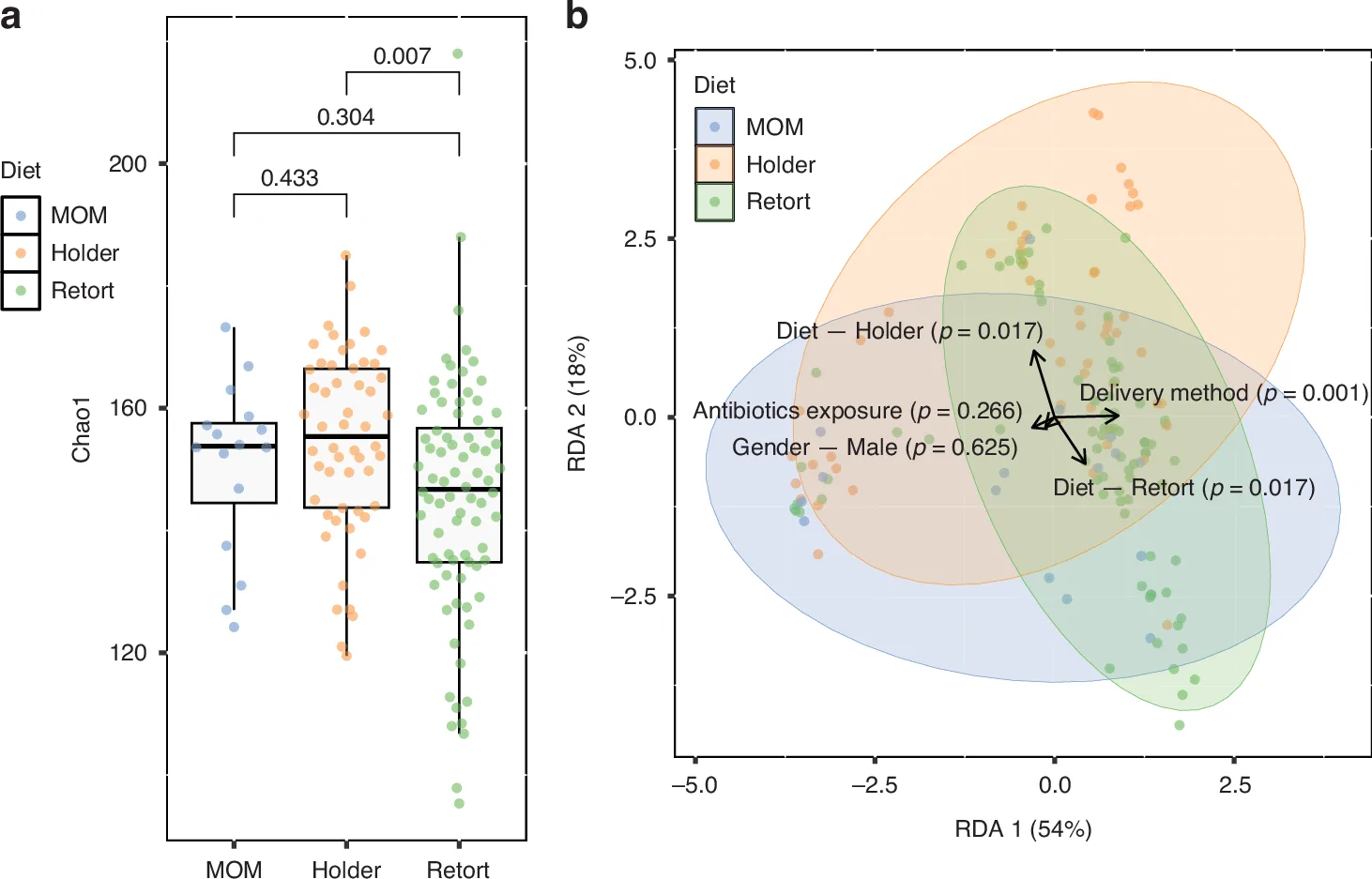
Alpha and beta diversity of the gut microbiome across diet groups. **a** Chao1 α diversity index comparing microbiome richness across diet groups: MOM, Holder, and Retort. The Holder group exhibited significantly higher Chao1 diversity compared to Retort (*p* = 0.007), while neither Holder nor Retort differed significantly from the MOM group. **b** Distance-based Redundancy Analysis (dbRDA) plot illustrating β diversity between groups while controlling for delivery method, antibiotic exposure, and sex. Diet, specifically Holder and Retort, significantly influenced microbiome composition (*p* = 0.017 for both). Delivery method also had a significant impact (*p* = 0.001), whereas antibiotic exposure (*p* = 0.266) and sex (*p* = 0.625) were not significant.

Microbiome diversity, measured by Chao1 α diversity, was significantly higher in the Holder group compared to the Retort group (*p* = 0.007). However, neither the Holder nor Retort groups showed a statistically significant difference from the MOM group (Fig. 1a). The evenness of species was low; therefore, the Shannon index did not show a statistically significant difference between Holder and Retort (*p* = 0.13), nor between these groups and the MOM cohort (*p* = 0.22–0.75).

To assess β diversity, we used distance-based redundancy analysis and PERMANOVA while controlling for variables including delivery method, antibiotic exposure, and sex. The PERMANOVA results indicate that diet variables, including Holder-pasteurized, Retort-pasteurized, and MOM, significantly influence gut microbiome composition (*p* = 0.017) (Fig. 1b). Mode of delivery also significantly influenced gut microbiome composition (*p* = 0.001) (Fig. 1b).

**Fig. 2:**
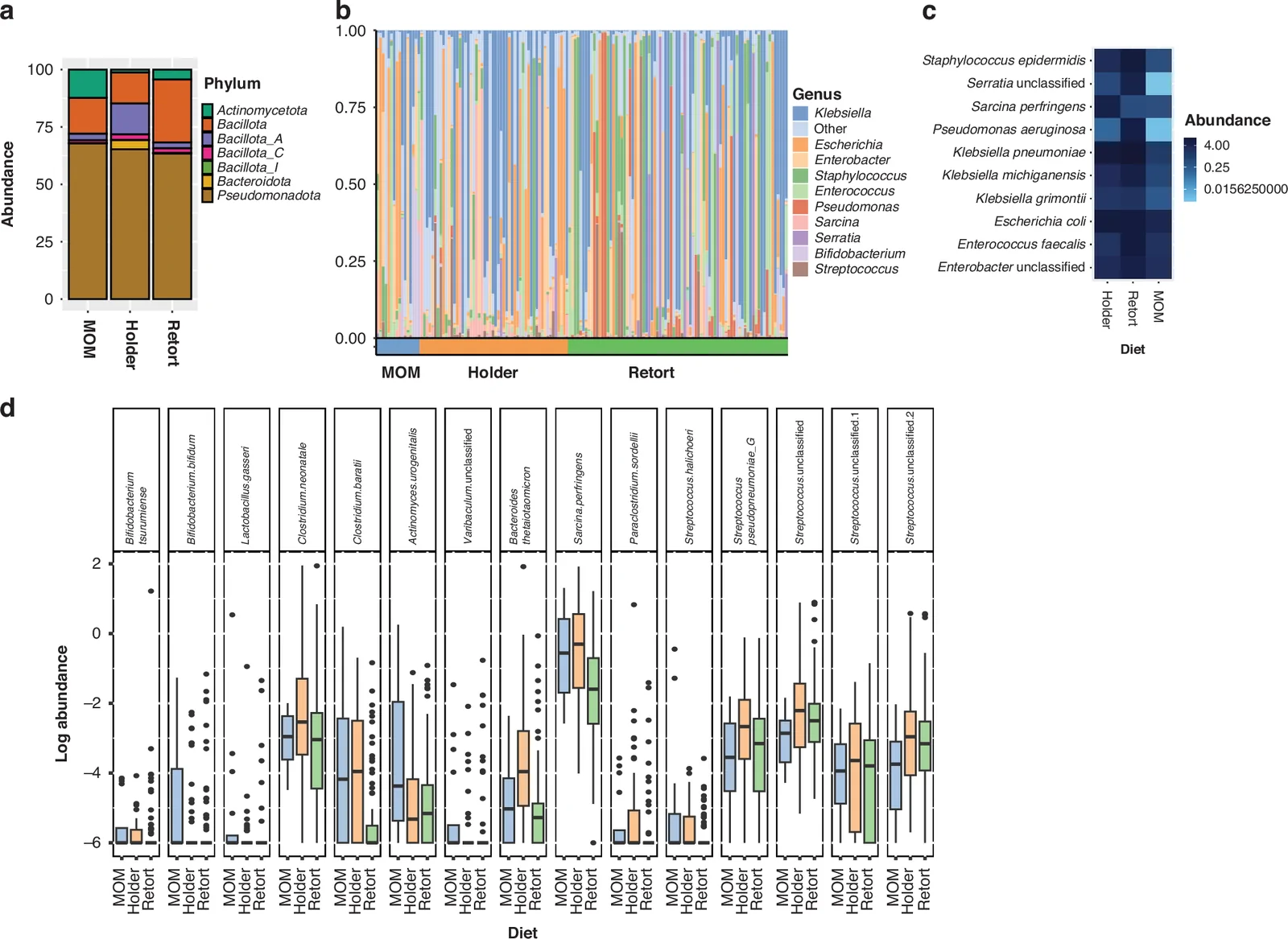
Taxonomic composition and differential abundance analysis of the gut microbiome across diet groups. **a** Relative abundance of bacterial phyla in MOM, Holder-, and Retort-pasteurized milk groups, highlighting differences in Actinomycetota, Bacillota, Bacteroidota, and Pseudomonadota. **b** The top 10 most abundant genera across all three groups, showing differences in microbial composition among diet groups. **c** The top 10 most abundant species across all three groups and a species heatmap illustrating relative abundance differences among Holder, Retort, and MOM. **d** Differential abundance analysis using ANCOMBC2 identified species significantly different between groups (*p* < 0.05), highlighting microbial shifts associated with diet.

When analyzing the relative abundance of organisms at the phylum, genus, and species levels, we observed distinct differences between groups. Infants fed with Retort-pasteurized MOM had a lower abundance of *Actinomycetota* and a higher abundance of *Bacillota* compared to the MOM-fed group (Fig. 2a). The Holder-pasteurized DHM-fed group exhibited the lowest *Actinomycetota* abundance but an increased presence of *Bacillota* A, *Bacillota* C, and *Bacteroidota* compared to MOM-fed infants (Fig. 2a).

At the genus level, the most abundant taxa across groups included *Klebsiella, Escherichia, Enterobacter, Staphylococcus, Enterococcus, Pseudomonas, Sarcina, Serratia, Bifidobacterium*, and *Streptococcus* (Fig. 2b). The top 10 most abundant species across all three groups were *Staphylococcus epidermidis, Enterobacter spp., Enterococcus faecalis, Escherichia coli, Klebsiella grimontii, Klebsiella michiganensis, Klebsiella pneumoniae, Pseudomonas aeruginosa, Sarcina perfringens*, and unclassified *Serratia*.

To identify species that differed significantly between infants fed MOM, Retort- or Holder-pasteurized DHM, we performed a differential abundance analysis using ANCOMBC2 while controlling for delivery method, antibiotic exposure, and sex. The species that exhibit higher abundance in infants fed Holder-pasteurized DHM compared to MOM include *Streptococcus pseudopneumoniae_G, Clostridium neonatale, Streptococcus oralis_BY*, and unclassified *Streptococcus* (Fig. 2d and Supplemental Table S1). Conversely, the species that show greater abundance in MOM-fed relative to Holder-pasteurized DHM-fed infants consisted of *Bifidobacterium tsurumiense, Streptococcus halichoeri, Actinomyces urogenitalis, Bifidobacterium bifidum*, an unclassified species of *Varibaculum*, and *Lactobacillus gasseri* (Fig. 2d and Supplemental Table S1).

In the comparison to MOM-fed infants, four species were less abundant in the Retort-pasteurized DHM-fed group: *Clostridium baratii, S. halichoeri, Varibaculum unclassified, and Paraclostridium sordellii* (Fig. 2d and Supplemental Table S2). When comparing Retort- to Holder-pasteurized DHM, three species were more abundant in the Holder compared to the Retort group: *Bacteroides thetaiotaomicron, S. perfringens, and P. sordellii* (Fig. 2d and Table S3). Multiple *Streptococcus unclassified* entries are shown separately in Fig. 2d due to either insufficient genomic resolution or limitations in classification algorithms and likely represent distinct species; therefore, they were not combined into a single group.

We did not measure a difference in short-term clinical outcomes between infants fed Holder- versus Retort-pasteurized DHM, there was no statistically significant difference in rates of NEC (14.8% vs 21.2%, *p* = 0.34), BPD (40.7% vs 38.8%, *p* = 0.785), IVH (25.9% vs 23.8%, *p* = 0.816), ROP (24% vs 34%, *p* = 0.367) or mortality (3.7% vs 2.5%, *p* = 0.411), respectively. Length of hospital stay was similar in the Holder-pasteurized DHM and Retort-pasteurized DHM groups (94.92 ± 85.81 days vs 79.09 ± 52.07 days, *p* = 0.56, respectively). The results were similar when including infants fed MOM, although the rate of ROP was statistically significantly lower in the MOM-fed group compared to both DHM cohorts (0% vs 24% and 34%, respectively, *p* = 0.009) (Table 2).

**Table 2:** Clinical outcomes

	Retort (*n* = 80)	Holder (*n* = 54)	MOM (*n* = 16)	*P*-value
NEC^a^ (%)	17 (21.2)	8 (14.8)	1(6.2)	0.340
BPD^b^ (%)	31 (38.8)	22 (40.7)	9 (56.2)	0.428
IVH^c^ (%)	19 (23.8)	14 (25.9)	3 (18.8)	0.888
ROP^d^ (%)	27 (33.8)	13 (24.1)	0 (0.0)	0.009
LoS^e^ (days)	79.09 ± 52.07	94.92 ± 85.81	92.56 ± 27.89	0.440
Mortality (%)	2 (2.5)	2 (3.7)	1 (6.2)	0.524

^a^Necrotizing enterocolitis.

^b^Bronchopulmonary dysplasia.

^c^Intraventricular hemorrhage.

^d^Retinopathy of prematurity.

^e^Length of stay.

**Fig. 3:**
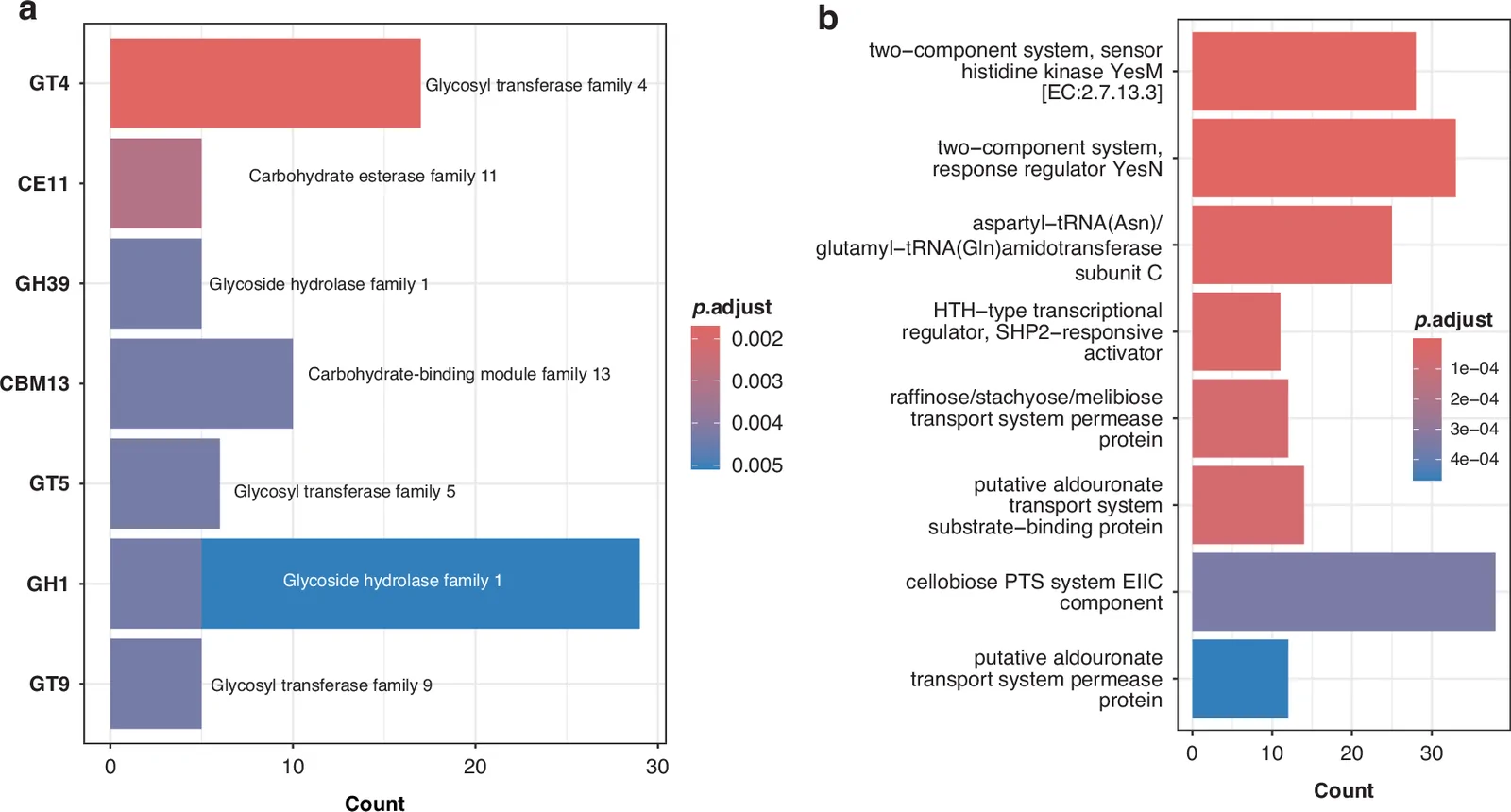
Functional enrichment analysis of the neonatal gut microbiome. **a** GO enrichment analysis of carbohydrate metabolism-related genes in Holder-pasteurized, Retort-pasteurized, and MOM-fed neonates. Holder-pasteurized milk-fed neonates exhibited significant enrichment of genes involved in carbohydrate metabolism, including glycosyl transferase (GT) and glycoside hydrolase (GH) families, carbohydrate-binding modules (CBM), and carbohydrate esterase (CE) enzymes. **b** Enrichment of transport and regulatory pathways in Holder milk-fed neonates. Genes associated with ABC transporters and phosphotransferase systems (PTS), including aldouronate and raffinose/stachyose/melibiose transporters, were significantly enriched. Two-component system regulators (*YesN/YesM*) and HTH-type transcriptional regulators were also overrepresented, suggesting microbial adaptation to Holder-pasteurized milk composition.

We performed GO enrichment analysis on species significantly more abundant in the Holder, Retort, and MOM groups (Fig. 2d). This analysis assessed the enrichment or over-representation of genes within species that showed significantly higher abundance in each group compared to the entire set of gut microbiome genes. GO overrepresentation analysis focused on the genes from ten species that were more abundant in Holder-pasteurized DHM-fed infants compared to MOM-fed and Retort-pasteurized DHM-fed patients (Supplemental Tables S1 and S3). The results indicate distinct functional profiles for the Holder group compared to MOM and Retort-pasteurized DHM-fed neonates (Fig. 3). In contrast, a similar analysis using the ten species more abundant in MOM-fed infants showed no significant GO enrichment functions. Functional profiles associated with carbohydrate metabolism and transport were significantly enriched in the stools of Holder-pasteurized DHM-fed infants (Fig. 3a). This included carbohydrate-active enzymes and several glycoside hydrolase (GH) families, including GH1 and GH39. Glycosyl transferase (GT) families, including GT4, GT5, and GT9, were predominant, indicating increased carbohydrate biosynthesis and degradation pathways in the Holder-pasteurized DHM-fed group. Carbohydrate-binding module (CBM13) and carbohydrate esterase (CE11) were also overrepresented, suggesting microbial adaptation to the carbohydrate composition in Holder-pasteurized milk fed infants. Similarly, several genes encoding ABC transporters and phosphotransferase systems (PTS) were enriched in Holder-pasteurized DHM-fed neonates (Fig. 3b). Specific transporters, including putative aldouronate transport systems, cellobiose PTS system components, and raffinose/stachyose/melibiose transporters, were significantly represented in this group. These findings suggest that microbial communities in Holder-pasteurized milk-fed infants utilize diverse sugar uptake mechanisms, distinct from those in MOM or Retort-pasteurized DHM-fed neonates. Two-component system regulators (e.g., YesN/YesM) and HTH-type transcriptional regulators were significantly enriched in the Holder-pasteurized DHM group. These regulators are involved in bacterial responses to environmental cues, further emphasizing microbial adaptation to the unique composition of Holder milk. Furthermore, we identified 186 unique antimicrobial resistance genes across all samples. However, there was no substantial difference in their overall abundance between the groups, except for the aminoglycoside 6′-N-acetyltransferase gene, which was significantly higher in Holder-pasteurized DHM-fed infants compared to MOM or Retort-pasteurized DHM-fed patients.

## Discussion

While MOM is the preferred nutrition source for premature infants,^1–5^, it is not always available. In this case, the American Academy of Pediatrics supports the use of DHM as an alternative.^5,39^ Although the protective effects of human milk on decreasing rates of NEC in premature infants are sustained despite pasteurization when compared to formula feeding,^40^ pasteurization has measurable effects on the quantity of protective factors in human milk.^11^ Compared to MOM, pasteurized DHM changes the intestinal gut microbiome of preterm infants.^41,42^ However, methods of DHM pasteurization vary, and to the best of our knowledge, we are the first to compare the gut microbiome between preterm infants fed Holder- versus Retort-pasteurized DHM.

We found differences in the gut microbiomes of infants fed Retort-pasteurized DHM compared to Holder-pasteurized DHM. Specifically, we found a higher relative abundance of *B. thetaiotaomicron* in the stool of Holder-pasteurized infants compared to the Retort-pasteurized DHM group. This gut bacterium was shown to be critical in regulating enteric neuronal and enteroendocrine cell populations.^42^ This observation indicates that the Holder diet may facilitate an increase in the prevalence of beneficial microbes. Conversely, Retort-pasteurized DHM fed preterm neonates exhibited lower alpha diversity and a reduced abundance of anaerobic bacteria, particularly *Clostridium* *spp*. Although lower bacterial diversity, lack of *Bifidobacteria*, and a reduced abundance of *Clostridia* have been identified as risk factors for NEC,^43^ we did not detect a significant effect on the rate of NEC in our study population. A much larger sample may be required to measure significant clinical outcomes such as NEC.

Retort pasteurization may contribute to an altered microbial landscape that could potentially increase NEC susceptibility compared to MOM. Clostridia play an essential role in gut development, including the production of short-chain fatty acids and the regulation of intestinal immune responses. The lower abundance of these beneficial anaerobes in the Retort-pasteurized DHM group raises concerns about the potential implications for gut health and immune maturation in preterm infants. Infants fed Holder-pasteurized DHM showed a significantly greater species diversity compared to infants fed Retort-pasteurized DHM, and overall, the microbiota of Holder-pasteurized DHM-fed infants was more similar to MOM-fed infants compared to Retort-pasteurized DHM-fed patients.

Functional enrichment analysis revealed significant differences in carbohydrate metabolism, transport systems, and regulatory pathways between the feeding groups, suggesting distinct microbial adaptations to Holder and Retort pasteurization. Holder-pasteurized milk-fed neonates exhibited increased representation of GH families and carbohydrate-binding modules, suggesting enhanced polysaccharide degradation capabilities. The enrichment of raffinose/stachyose/melibiose transport systems indicates microbial adaptation to specific oligosaccharides present in this feeding group. Additionally, the enrichment of two-component signal transduction systems suggests heightened microbial metabolic regulation in response to the composition of Holder-pasteurized milk. These findings suggest that microbial communities dynamically adjust their carbohydrate metabolism and regulatory networks depending on milk type, influencing overall microbiome composition and metabolic activity. Future studies should explore the direct impact of these functional adaptations on neonatal health and development.

The only clinical outcomes that were statistically significantly different between the groups in our study was a lower rate of ROP in preterm infants fed MOM, even though infants in the MOM group were more premature. While feeding MOM was found to be protective against ROP compared to formula,^44^ the protective effect of DHM is less clear.^45^ Although our sample size is small, our data suggest that DHM is inferior to MOM regarding ROP prevention. While we did not see differences in other short-term outcomes, the observed microbial shifts could have implications for long-term health, which requires further investigation. Future research should focus on functional metagenomics, metabolomics, and host-microbiome interactions to better understand the long-term impact of pasteurization methods on preterm infant health. In addition, future studies should compare intestinal permeability in gestational age- and age-matched infants being fed different forms of DHM versus MOM, as the type of feeding may affect the development of the intestinal barrier.^46^

Our study has several limitations. First, the baseline characteristics of our cohorts revealed a higher C-section rate in the Retort cohort compared to infants fed Holder-pasteurized milk. However, stool samples for our analysis were collected at 4 weeks, by which time, the gut microbiome between infants born via vaginal delivery and those born via C-section is more similar.^47^ Second, while exposure prior to sample collection was similar in the Retort and Holder cohorts (*p* = 0.37), we cannot rule out that differences in diversity and microbiome composition were influenced more by antibiotics than by type of nutrition. Maternal antibiotic exposure for latency or for wound infection prophylaxis may play a role and was likely present in most patients. However, at 4 weeks of age, it’s impact on the infant’s gut microbiome is less likely measurable. In addition, although antibiotic exposure was statistically significantly lower in the MOM group compared to either DHM group, microbiota composition between the Holder and MOM groups were similar, suggesting that type of feeding had a stronger impact on the intestinal microbiome than antibiotic exposure. Third, analysis of a single stool sample cannot capture longitudinal changes in the infant gut microbiome. While we noticed a trend towards improved outcomes in the Holder versus Retort group, we found no statistically significant differences in selected short-term clinical outcomes between our cohorts. This may be due to a low sample size. Additionally, there may have been differences in other outcomes that we did not analyze. Lastly, we do not know which specific compounds in DHM are affected by each pasteurization method. For example, pasteurization reduces levels of hormones such as insulin and leptin in human milk, which may influence the composition of the intestinal microbiome.^15,48^

In conclusion, our data suggest that the type of DHM pasteurization affects the composition of the intestinal microbiome and its functional properties in preterm infants. Holder pasteurization was associated with a richer microbiome and bacterial abundance that was closer to the intestinal microbiome of infants fed MOM. Although future studies should include larger sample sizes and longitudinal data, our results suggest microbial benefits of Holder-pasteurized DHM over Retort-pasteurized DHM for preterm infants.

## Supplementary information

Supplemental Data

## Data Availability

Raw shotgun sequencing data will be made publicly available in the NCBI Sequence Read Archive (SRA) under BioProject accession number **PRJNA1312114** upon publication of this manuscript.
