# Supplementary material for: Impact of donor human milk pasteurization methods on the gut microbiome of preterm infants
Source: Pediatr Res. 2025 Sep 16;100(3):1414–20. doi: 10.1038/s41390-025-04386-2 (PMC13672114; doi:10.1038/s41390-025-04386-2)
Supplement: Supplementary file 1 [file 41390_2025_4386_MOESM1_ESM.pdf]

Supplemental Table 1. Differentially abundant species comparing feeding Holder-pasteurized DHM versus MOM. A positive log fold change (lfc) denotes higher abundance in Holder-pasteurized DHM fed compared to MOM fed infants, and a negative number means higher abundance in MOM fed compared to Holder-pasteurized DHM fed infants.

**Taxon**	**lfc (Holder vs MOM)**	**q-value**
Streptococcus pseudopneumoniae_G	3.08	0.00
Clostridium neonatale	2.85	0.02
Streptococcus_unclassified	2.70	0.00
Streptococcus_unclassified	2.57	0.02
Streptococcus_unclassified	2.51	0.01
Streptococcus_unclassified	2.28	0.04
Streptococcus oralis_BY	2.17	0.05
Bifidobacterium tsurumiense	-2.53	0.00
Streptococcus halichoeri	-2.60	0.04
Actinomyces urogenitalis	-3.08	0.02
Bifidobacterium bifidum	-3.25	0.02
Varibaculum_unclassified	-3.44	0.00
Lactobacillus gasseri	-3.45	0.01

Supplemental Table 2. Differentially abundant species comparing feeding Retort-pasteurized DHM versus MOM. A negative log fold change (lfc) denotes higher abundance in MOM fed compared to Retort-pasteurized DHM fed infants.

**Taxon**	**lfc (Retort vs MOM)**	**q-value**
Clostridium baratii	-4.23	0.00
Streptococcus halichoeri	-3.56	0.00
Varibaculum_unclassified	-2.77	0.00
Paraclostridium sordellii	-2.07	0.02

Supplemental Table 3. Differentially abundant species comparing feeding Holder- versus Retort-pasteurized DHM. A positive log fold change (lfc) denotes higher abundance in Holder- compared to Retort-pasteurized DHM fed infants.

**Taxon**	**lfc (Holder vs Retort)**	**q-value**
Bacteroides thetaiotaomicron	3.12	0.01
Sarcina perfringens	3.04	0.02
Paraclostridium sordellii	2.32	0.05
